# Epigenetic silencing of miR-493 increases the resistance to cisplatin in lung cancer by targeting tongue cancer resistance-related protein 1(TCRP1)

**DOI:** 10.1186/s13046-017-0582-5

**Published:** 2017-08-31

**Authors:** Yixue Gu, Zhijie Zhang, Jiang Yin, Jiahui Ye, Yin Song, Hao Liu, Yan Xiong, Minying Lu, Guopei Zheng, Zhimin He

**Affiliations:** 1 Affiliated Cancer Hospital & Institute of Guangzhou Medical University, Guangzhou, Guangdong, 510095 China; 2 Department of Pharmacology, Guangzhou Institute of Snake Venom Research, School of Pharmaceutical Sciences, Guangzhou Medical University, Guangzhou, Guangdong 511436 China

**Keywords:** Lung cancer, Cisplatin miR-493, TCRP1

## Abstract

**Background:**

The potential mechanisms regarding how methylation of microRNA(miRNA) CpG Island could regulate cancer cell chemo-resistance remains unclear. This study aims to explore the epigenetic dysregulation mechanism of miRNA-493 and the ability to modulate lung cancer cell chemotherapy resistance.

**Methods:**

Real-time quantitative PCR (qRT-PCR) and In situ hybridization (ISH) were used to analyze the expression of miR-493 in lung cancer cell lines and tumor tissue, respectively. Bisulfite sequencing PCR (BSP) was used to exam the promoter CpG Island of miR-493. The effect of miR-493 on chemosensitivity was evaluated by cell viability assays, apoptosis assays and in vivo experiment. The DNA damage was measured by γ-H2AX immunofluorescence. Luciferase reporter assay was used to assess the target genes of miR-493. Expression of target proteins and downstream molecules were analyzed by Western blot.

**Results:**

miR-493 is silenced in resistant lung cancer cell due to the aberrant DNA methylation. Enforced expression of miR-493 in lung cancer cells promotes chemotherapy sensitivity to cisplatin through impairing the DNA damage repair and increasing the cells apoptosis in vitro and in vivo. Furthermore, we identify that TCRP1 is a direct functional target of miR-493. Ectopic expression of TCRP1 attenuated increased apoptosis in miR-493-overexpressing lung cancer cells upon cisplatin treatment. Meanwhile, miR-493 level is negatively correlated with TCRP1 expression in lung cancer patients and TCRP1 expression were correlated with poor survival.

****Conclusion**s:**

Our results highlight that hyper-methylation of miR-493CpG island might play important roles in the development of lung cancer chemo-resistance by targeting TCRP1, which might be used as a potential therapeutic target in preventing the chemo-resistance of lung cancer.

## Background

Non-small cell lung cancer (NSCLC) accounts for 85% of all lung cancer cases and is the leading cause of cancer-related deaths worldwide with a 5-year survival of approximately 15% [1]. Drug resistance is a barrier for curative lung cancer therapies due in part to the molecular heterogeneity of tumors. A major contributor to intratumor heterogeneity is DNA methylation and repressive chromatin states that are also recognized to play major roles in lung cancer therapy resistance [2, 3]. Importantly, gene-specific hypermethylation is an early and likely an initiating event in the process of tumor development [4–6].

Epigenetic regulation involving DNA methylation is a heritable and enzyme-induced modification in human, which modulate the expression of target mRNA without direct changing of the DNA sequences. Epigenetic modification of mRNA CpG islands has been widely reported to down-regulate the target mRNA expression in cancer-related malignant phenotypes [7, 8]. The hypermethylation of promoter CpG Island affects not only tumor suppressive mRNAs, but also tumor suppressive miRNAs. The hyper-methylation in the CpG islands of miRNA promoter can silence the expression of tumor-suppressive miRNAs or drug sensitizing miRNAs, resulting in oncogenic or chemo-resistant phenotypes in cancers. Some tumor suppressive miRNAs such as miR-34a and miR-375 have been reported to be silenced by the hypermethylation of promoter regions and play important roles in the related cancers [9, 10]. Recent studies by Xi et al. [11] support this supposition by demonstrating that miR-487b is epigenetically silenced and involved in the pathogenesis of lung cancer.

In our previous study, we established a cisplatin-resistant cell line (A549/DDP) derived from A549 lung cancer cells by stepwise selection using cisplatin (cDDP). And it exhibited a stable growth pattern in the medium with indicated concentration cisplatin. Recently, we found that miR-493, which was characterized as a tumor-suppressive miRNA in some cancers including colon cancer, bladder cancer, etc. [12–14], was dysregulated in lung cancer cell lines, especially in cisplatin resistant cells [15]. But it is unclear that the silenced miR-493 is a causal factor for resistance to cisplatin or an accompanied phenomenon in lung cancer cell lines. Meanwhile, the specific conditions and mechanisms by which miRNA-493 gene is silenced are not completely described. Otherwise, previously, a novel resistance relative gene, tongue cancer resistance-associated protein 1 (TCRP1) was cloned by our lab from the tongue cancer multi-drug resistance cell line Tca8113/Pingyangmycin (Tca8113/PYM), which mediated a specific resistance to cDDP part due to activation of the PI3K/Akt/NF-κB signaling pathway [16–19]. Interestingly, in preliminary experiments we found expression of TCRP1 was negative relative to miR-493 in A549 and its resistant subclones A549/DDP cells. Then we supposed that epigenetic silencing of miR-493 could increase the resistance to cisplatin by targeting TCRP1. In this study, we explore the promoter region of miR-493 methylation status in lung cancer cell lines. Furthermore, we identify whether miR-493 is a critical miRNA, which regulates lung cancer cells sensitivity to cisplatin by targeting potential resistant relative gene TCRP1.

## Methods

### Cell culture and tissue specimens

Human lung cancer cell lines (H1975, A549, H1299, H460) were obtained from and maintained as recommended by the American Type Culture Collection (ATCC, Manassas,VA, USA).The human lung cancer cell line 95D was obtained from the Cell Bank of the Chinese Academy of Sciences (Shanghai, China). The stable cDDP resistant cell line A549/DDP was previously established in our lab. Above cell lines were maintained in RPMI-1640 medium (Hyclone, Logan, UT, USA), supplemented with 10% fetal bovine serum (Hyclone) at 37 °C in a humidified atmosphere containing 5% CO_2_. To maintain the resistance phenotype, 3uM cDDP was added to the culture media of A549/DDP cells uninterruptedly. cDDP was purchased from Sigma-Aldrich (Sigma-Aldrich, US).

All lung cancer tissue specimens were collected via surgical resection from patients diagnosed between March 2007 and March 2013 at the Affiliated Tumor Hospital of Guangzhou Medical University (Guangzhou, Guangdong, China). Written informed consent was obtained from all study participants. The study protocol was approved by the Ethics Committee of Guangzhou Medical University. Overall survival was computed from the day of surgery to the day of death or of last follow-up.

### 5-Aza-dC treatment and Bisulfite sequencing

For 5-Aza-dC treatment, cells were cultured with serial diluted 5-Aza-dC (0-5 μM) for 48 h, and then were harvested for the detection of miRNA expression. Bisulfite-converted genomic DNA (100 ng) (Zymo Research, Irvine, CA, US) was amplified by using bisulfite sequencing primers. The PCR products were cloned and 5 individual clones were sequenced.

For bisulfite sequencing PCR (BSP), Genomic DNA was extracted from cells using TRIZOL (Invitrogen, Carlsbad, CA, USA), and was then subjected to bisulfite conversion using the EZ DNA Methylation-Gold Kit (Zymo Research) according to the manufacturer’s instructions. The bisulfite-converted genomic DNA was used for the methylation analysis of miR-493 with the predicted methylation primers, which were designed according to the online primers program “MethPrimer” (http://www.urogene.org/cgi-bin/methprimer/methprimer.cgi). The primers used for BSP are as follows: MSP1, 5′-GAATTTTAGGATTAGATGGGGTTTT-3′ (forward), 5′-TACAATACAAAAACAAACATTTCCC-3′ (reverse). MSP2, miR-493–F 5′ TGTGATTGGAATGGAAATTTAATTT, miR-493–R 5′ ACTATCCTACACTCCCCTACCCTAC 3′. The amplified fragments were cloned into the pGEMT Easy vector (Promega, Madison, WI, US), and five clones were randomly selected for bisulfite sequencing.

### Reverse transcriptase (RT)-PCR and qRT-PCR

miRNAs from cultured cells were isolated and purified with the miRNA isolation system (Exiqon, Vedbaek Denmark). cDNA was generated with the miScript II RT Kit (QIAGEN, Hilton, Germany), and quantitative real-time PCR (qRT-PCR) was performed by using the miScript SYBR Green PCR Kit (QIAGEN) following the manufacturer’s instructions. The miRNA sequence-specific RT-PCR primers and the endogenous control RNU6 were purchased from Ribobio co., Ltd. (Ribobio, Guangzhou, China). The total RNA was extracted according to the Trizol protocol, and cDNAs from the mRNAs were synthesized with the first-strand synthesis system (Thermo Scientific, Glen Brunie, MA, USA). Realtime PCR was carried out according to standard protocols using an ABI 7500 with SYBR Green detection (Applied Biosystems, Foster, CA, USA). GAPDH was used as an internal control. The primer sequences of TCRP1 were defined as follows: Forward primer: 5′-GAACTCGTCTTCCTGTGGCA-3′ Reverse primer: 5′-GGGGTGGAGCAGTGTTACTC-3′. The primer sequences of GAPDH were defined as follows: Forward primer: 5′-CCACATCGCTCAGACACCAT-3′; Reverse primer: 5′-TGACAAGCTTCCCGTTCTCA-3′. Real-time PCR was performed using a standard protocol for the SYBR Green PCR kit (Toyobo,Osaka, Japan). The 2-ΔΔCt method was used to determine the relative quantities of gene expression. Each sample was analyzed in triplicate.

### Western blot

Whole-cell lysates were prepared with RIPA lysis buffer (50 mM Tris–Cl, pH 7.4, 150 mM NaCl, 1% Triton X-100, 1% sodium deoxycholate, 0.1% SDS, 1 mM sodium orthovanadate, 10 mM sodium fluoride, 1% protease inhibitor cocktail). The proteins were separated by SDS-PAGE gel, and Western blot was performed as described previously [13]. Antibodies against TCRP1, Akt, p-Akt and β-actin were purchased from Santa Cruz Biotechnology (Santa Cruz, US),. Antibodies against GSK3βand p-GSK3β(ser9) were purchased from cell signaling Technology (Cell signaling, US).

### 3’UTR construction and Luciferase reporter assay

A pmirGLO Dual-Luciferase miRNA target expression vector was used for the 3′-UTR luciferase assays (Promega). The target genes of miRNA-493 were selected based on the target scan algorithms [microRNA.org (http://www.microrna.org/microrna/home.do) and TargetScan (http://www.targetscan.org/)]. The primer sequences used were defined as follows: TCRP1 forward primer: GCCTCGAG CAGGACACCCAGCCCAGACA, the reverse primer: CTAGCGGCCGCCGCTCCTCCCTTCTTCCCTCT. Mutant TCRP1 forward primer: GAGGTGAGTGGGCGCTCCTCCCTTCTTCCCT, Mutant TCRP1 reverses primer: CCTGCCCACATCAGCAGCACCACCCAGCAA. Bold indicates the XhoI (CTCGAG), and underline indicates the NotI internal site. For the 3′-UTR luciferase assay, 293 T cells were cotransfected with hsa-miR-493 and pmirGLO Dual-Luciferase miRNA target with hsa-miR-493 and pmirGLO Dual-Luciferase miRNA target expression vectors with wild-type or mutant target sequence using Lipofectamine 2000.The luciferase assay was conducted using the Dual-Luciferase Reporter Assay System (Promega) 48 h after transfection. The data are presented as the mean value ± SD for triplicate experiments and compared with the level of wild-type sequence vectors obtained in mutant-type sequence vector transfected cells that are normalized to 100%.

### Lentivirus production and infection

To construct a vector expressing miR-493, the precursor sequence of miR-493 (MI0003132) was synthesized, annealed and then inserted into the BamHI–HindIII fragment of the pGCIL3 vector (GeneCopoeia, Inc., Rockville, MD USA).The lentivirus plasmids were co-transfected with pLP1, pLP2, and pLP/VSVG (GeneCopoeia) into 293 T cells, and the virus-containing supernatants were prepared according to manufacturer’s instructions. For lentivirus infection, the cells were incubated with virus-containing supernatants in the presence of 6 mg/ml polybrene. The infected cells were selected in the presence of 2 mg/ml puromycin to generate two paired stable monoclonal cell lines (a stable cell line expressing miR-493, A549/miR-493, A549/DDP/miR-493 and their control stable cell line, A549/control or A549/DDP/control). Flow cytometry analyses were performed on cells infected with GFP expressing viruses for miRNA expression to confirm that 90% of cells were infected.

### MTS assay

The CellTiter 96 AQueous One Solution Cell Proliferation Assay kit (Promega, Madison, WI, USA) was used to determine the sensitivity of cells to cDDP. Briefly, cells were seeded in 96-well plates at a density of 4 × 10^3^ cells/well (0.2 ml/well) for 24 h before use. The culture medium was replaced with fresh medium containing cDDP at different concentrations and cells were then incubated for a further 72 h. Then, MTS (0.02 ml/well) was added. After further 2 h incubation, the absorbance at 490 nm was recorded for each well on the BioTek Synergy. The absorbance represented the cell number and was used for the plotting of dose–cell number curves.

### Immunofluorescence microscopy

Cells were seeded on coverslips. After indicated treatment, cells were washed with PBS and fixed in 4% paraformaldehyde for 15 min. Cells were permeabilized using 0.25% Triton X-100 solution for 5 min and then blocked in 5% bovine serum albumin for 1 h at room temperature. Coverslips were incubated with anti-γ-H2AX antibody (purchased from Millipore 1:500) overnight at 4 °C. After washed with PBS, the cells were incubated with Alexa Fluor 488 donkey anti-mouse IgG (purchased from Invitrogen, 1:1000) for 1 h. The coverslips were washed with PBS and mounted onto slides using proLong gold antifade reagent with DAPI (Life Technologies, US). Slides were visualized on a microscopy system.

### Flow cytometry analysis for cell apoptosis

The cells (1 × 10^6^) were digested with a trypsin solution, then harvested and washed twice with cold PBS. The washed cells were rinsed twice with PBS, and re-suspended in a propidium iodine solution comprising containing 40 μg/mL propidium iodine and 100 lg/mL RNaseA (Sigma-Aldrich) in PBS without calcium and magnesium, then incubated at 37 °C for 30 min in the dark. The stained cells were passed through a nylon mesh sieve to remove cell clumps, and then analyzed with FACScan flow cytometer and the CELL QUEST analysis software (Becton Dickinson, San Jose, CA, USA). The flow cytometry analysis was repeated three times.

### In vivo tumorigenesis assays

BALB/c-nude mice (female, 3-4 weeks of age, 18-20 g) were purchased from the Center of Experimental Animal of Guangdong province. All experimental procedures were approved by the Institutional Animal Care and Use Committee of Guangzhou Medical University. The BALB/c nude mice were randomly divided into 2 groups (each group contain 12 mice). A549 or A549/DDP cells (1 × 10^7^/site)were inoculated subcutaneously in the dorsal flanks of nude mice and developed into solid tumors in 7-10 days after injection. When the maximum tumor diameter exceeds 6um, each group were respectively treatment with 2 mg/kg cisplatin or equal volumes saline by intraperitoneally injection every other day for 4 weeks. Tumor sizes were measured with caliper every three days and calculated by the formula V = (L × W^2^)/2. Thirty days after tumor implantation, the mice were sacrificed; the subcutaneous tumors were removed and weighed. Tumors were fixed in formalin and embedded in paraffin using the routine method. Serial 6.0 μm sections were cut and subjected to H&E stained with Mayer’s hematoxylin solution.

### TCRP1-expressing vector

Full-length TCRP1 cDNA entirely lacking the 3′-UTR was purchased from GeneCopeia (Rockville, MD, USA) and subcloned into the eukaryotic expression vector pcDNA3.1(+) (Invitrogen). The empty pcDNA3.1 (+) vector was used as a negative control.

### miRNA in situ hybridizations (ISH) assay

The miR-493 expression in lung cancer samples and nude mice tumor tissues were detected by ISH with kit from Exiqon (Vedbaek Denmark) according to the manufacturer’s instructions. Briefly, the sections were dried at 65 °C for 3 h and then deparaffinized in xylene and ethanol at room temperature (RT) followed with 10 min incubation with proteinase-k at 37 °C. After dehydration in ethanol, sections were hybridizated with 40 nM double-DIG LNA™ miR-493 probe 55 °C for 1 h. After wash in SSC buffer at hybridization temperature and incubation with blocking solution for 15 min, the anti-DIG reagent sheep anti-DIG-AP (Roche, Mannheim, Germany) was applied and incubated for 60 min at RT. After wash in PBST, the sections were incubated with AP substrate NBT-BCIP (Roche) for 2 h at 30 °C and incubated in KTBT buffer to stop reaction. Then the nuclear counter stain Nuclear Fast Red™ (Vector labs, Burlingame, CA) was applied for 1 min for nuclear counter staining, and slides were rinsed in tap water for 10 min. After dehydrated in ethanol and mounted, the sections were investigated and analyzed under microcopy.

### Immunohistochemistry

Immunohistochemistry was performed on formalin-fixed paraffin-embedded tissue using anti-TCRP1 antibody (Sigma-Aldrich) and Ki67 antibody (Abcam Technology, Us) and the standard streptavidin–peroxidase staining method (Biotin-Streptavidin (ABC) IHC detection kits, Abcam Inc., US). Immunohistochemistry results were scored according to the intensity (0–4) and percentage of positive cells scores 0: no staining; 1: 10% positive cells; 2: 11–50% positive cells; 3: 51–75% positive cells; 4: .75% positive cells. The relative expression was obtained by multiplying the intensity by the percentage.

### Statistical analysis

The data are expressed as the mean ± standard error of the mean (SEM) from at least three independent experiments. The values for luciferase activity assays were obtained from three independent experiments performed in duplicate. Unless otherwise noted, the differences between groups were analyzed using Student’s t test when only two groups were compared or using a one-way ANOVA when more than two groups were compared. All statistical tests were two-sided. The differences were considered statistically significant at *P* < 0.05. All analyses were performed using SPSS software (SPSS Inc., Chicago, IL).

## Results

### miR-493 is silenced in resistant lung cancer cell due to the aberrant DNA methylation.

**Fig. 1:**
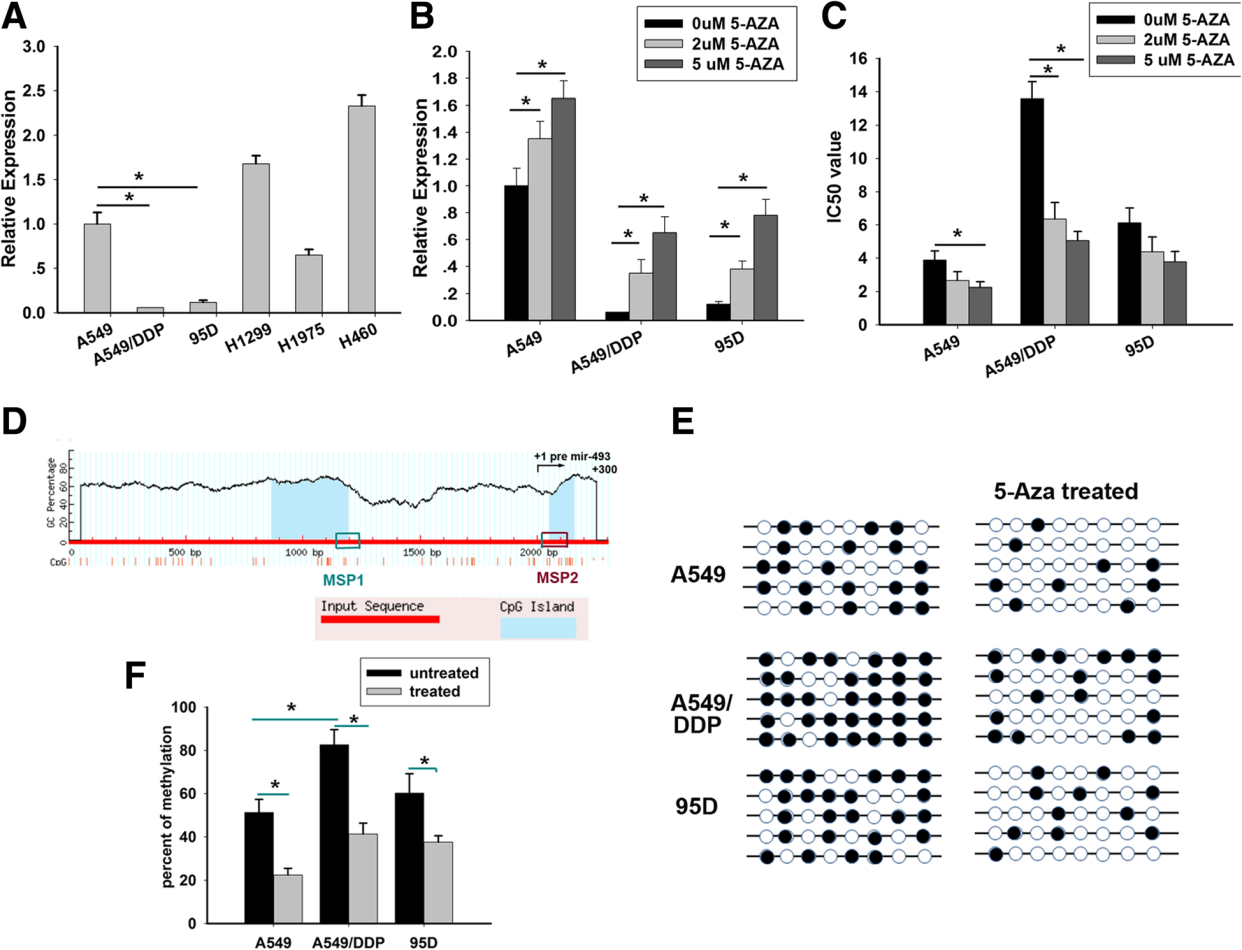
miR-493 was down-regulated due to the aberrant DNA hype-methylation in lung cancer cell lines. **a** Real-time PCR was used to test the miR-493 expression in a panel of lung cancer cell lines including A549, A549/DDP and 95D. Each experiment was independently repeated at least 3 times. **b** MiR-493 expression in A549, A549/DDP and 95D cell lines after treatment of 2 μM and 5 μM 5-AZA-dC was determined by real-time PCR and the relative miR-493 expression value was indicated. **c** IC50 values of cells to DDP calculated from MTT assays showing the effects of 5-AZA-dC on sensitivity to cisplatin in A 549, A549/DDP and 95D cells. Each experiment was independently repeated at least 3 times. Error bars correspond to the mean ± SD. (*p < 0.05). **d** Schematic map containing CpG islands (shaded area) of the miR-493 upstream 2Kb- − +300 bp region, as adapted from the MethPrimer website (upper panel), indicating the transcription start site (+1), Primers for methylation-specific PCR (MSP) and quantitative pyrosequencing were designed to study that CpG island. **e** Bisulfite sequencing analysis was performed on miR-493 (+54 to +156) in lung cancer cells. Each horizontal row represents a single clone; the methylation percentages of 5 individual clones were indicated. The white and black circles represent the unmethylated and methylated CpG sites, respectively. **f** The methylation percentage of each cell line was shown. Error bars correspond to the mean ± SD. (*p < 0.05)

It was previously found that the miR-493 was dysregulated in a cisplatin-resistant lung cancer cell line [15]. However, it is unclear whether the silenced miR-493 is a causal factor for resistance to cisplatin or not. Here, we first checked the expression of miR-493 using real time-PCR analysis in a panel of lung cancer cells. The data indicated that the expression of miR-493 was dramatically down-regulated in A549/DDP cells comparing with parent cell A549 (Fig. 1a). Furthermore, in order to test whether the aberrant down-expression of miR-493 was due to hypermethylation in lung cancer cell lines, we treated the lung cancer cell lines A549, A549/DDP and 95D with de-methylation reagent 5-AZA-dC, a DNA methyltransferase inhibitor. The data indicated that the expression miR-493 was significantly restored after treatment with 5-AZA-dC (Fig. 1b), suggesting that this miRNA is probably silenced by DNA methylation in lung cancer cells. Meanwhile, Using MTT, we found the IC50 values to cisplatin were decreased after 5-AZA-dC treatment in lung cancer lines (Fig. 1c), especially in resistant lung cancer cell A549/DDP, indicating that aberrant expression of miR-493 might involve to resistant abilities.

### A hyper-methylated CpG island in miR-493 contributed to epigenetic silence in lung cancer cell lines

Given the downregulation of miR-493 in resistant lung cancer cell lines, we sought to examine the possible regulation of miR-493 by DNA methylation. The miR-493 genomic locus contains 2 CpG islands that overlap with the miR-493-coding sequence and extends upstream into the miR-493 promoter region (Fig. 1d). To test if the methylation status of these CpG islands changes in resistant lung cancer cell lines, we examined the 3 lung cancer cell lines, to assess the extent of CpG methylation within miR-493 by bisulphite sequencing. We measured two distinct regions, named bisulphite sequencing 1 and bisulphite sequencing 2, within the CpG Island (Fig. 1 d, e). Region 1 was found to have no difference in methylation degree in A549/DDP and 95D cells comparing with A549 cell line (data not shown). Analysis of the second region displayed a significant higher degree of methylation in resistant cell lines (81.6% in A549/DDP, 62.3% in 95D) than sensitive cell line A549 (47.8%) (Fig. 1e, f). Furthermore, under the treatment of 5-AZA-dC (2 μM), the methylation percent of A549/DDP and 95D cells were remarkable decreased in treated groups compared with the control group (Fig. 1e,f). Together our analyses strongly pointed to a significantly higher rate of CpG methylation within the miR-493 locus in resistant lung cancer cell line versus sensitive cells.

### miR-493 modulates chemosensitivity in lung cancer cells in vitro and in vivo

**Fig. 2:**
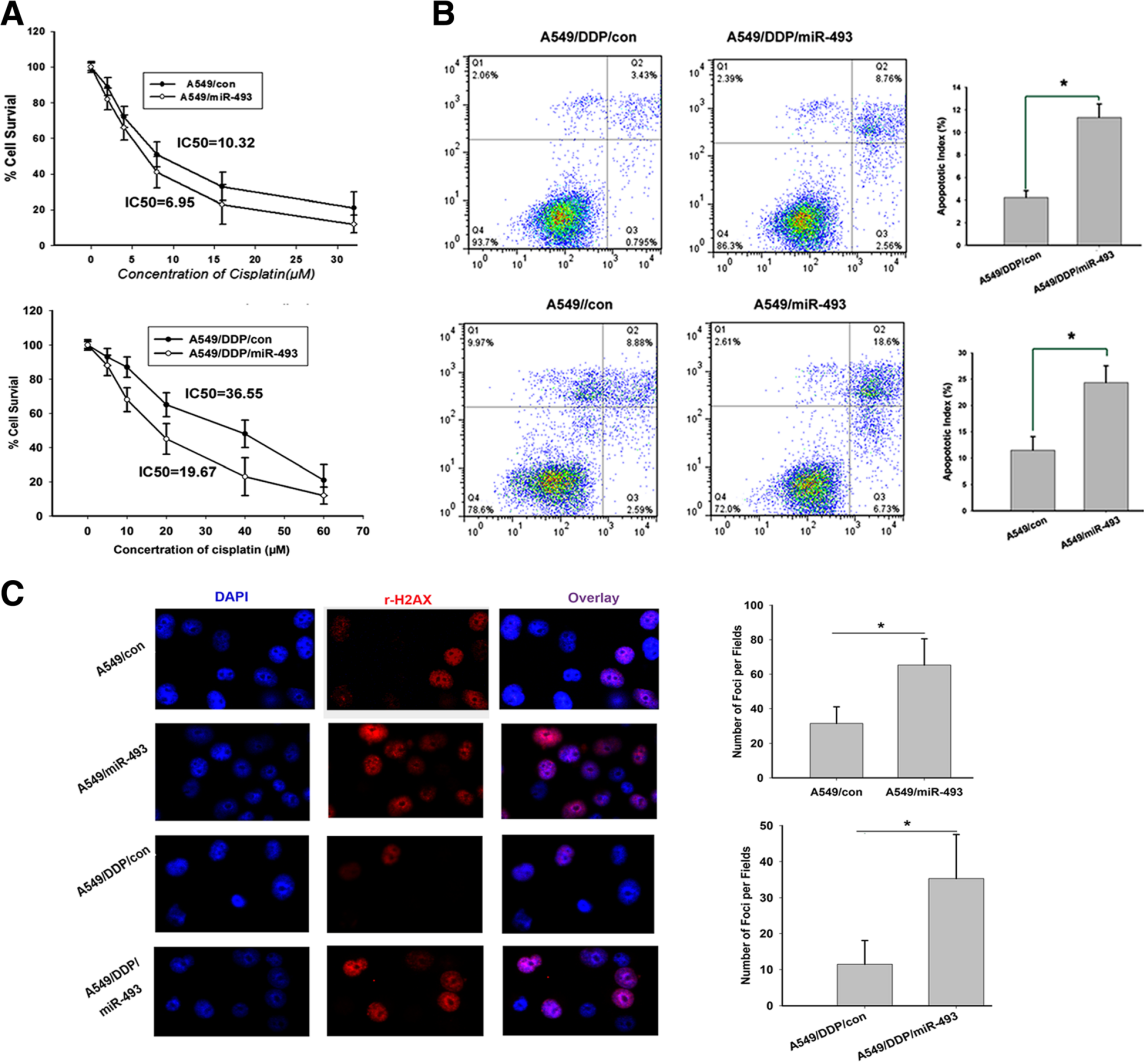
miR-493 increases the sensitivity of lung cancer cells to cisplatin in vitro. **a** miR-493 overexpression increases the sensitivity of A549 and A549/DDP cells to cisplatin. The IC50 of miR-493 overexpressed lung cancer cells was significantly lower than that of the control (*P* < 0.01). **b** miR-493 overexpression enhances cisplatin-induced apoptosis. A549 and A549/DDP cells were treated with 5 μg/mL cisplatin for 24 h. Cell apoptotic death events were monitored by Annexin V/PI staining and flow cytometry assays. Each experiment was independently repeated at least 3 times. Error bars correspond to the mean ± SD. (*p < 0.01). **c** miR-493 enhances DDP-induced DNA damage in lung cancer cells. Immunofluorescence staining was performed to examine γ-H2AX foci formation. A549 and A549/DDP cells were treated with 5 μg/mL cisplatin for 24 h. γ-H2AX foci formation was detected by fluorescence microscope. γ-H2AX foci were counted in 5 random 200× micro fields and categorized into the indicated groups. Representative images of the assays are shown. Original magnification: ×200.Significant differences are indicated, (*, *P* < 0.05)

**Fig. 3:**
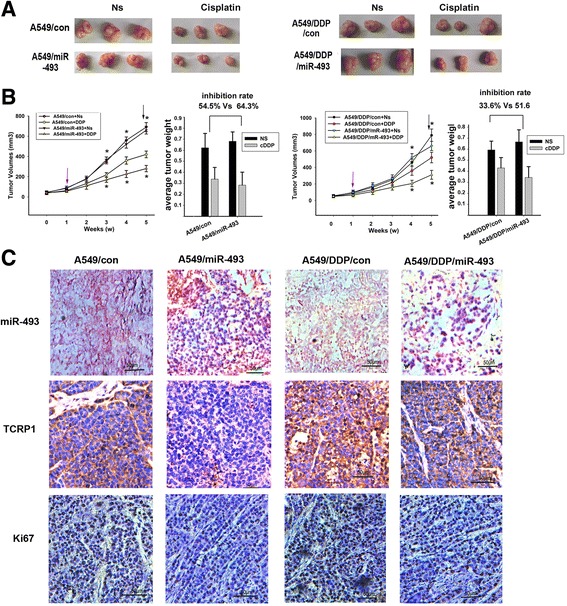
The effect of miR-493 on the sensitivity of lung cancer cells to cisplatin in nude mouse xenograft model. **a** and **b**, A549 and A549/DDP cells stably expressing miR-493 or the control was injected subcutaneously into the right flank of each nude mouse. The tumor growth curves and tumor inhibited rate were shown. The arrows indicated sites were the start treatment time with cisplatin or saline. Significant differences are indicated, (*, *P* < 0.05). **c** Representative images show ISH (red staining as nucleus and blue staining as miR-493 level) staining of the miR-493 tumors (×200) generated in nude mice and immunohistochemistry staining using TCRP1 and Ki67 antibodies on these tumors (×200)

To investigate whether the reduction of miR-493 played a causal role in the development of drug resistance, stable ectopic expression cell subsets A549/miR-493 and A549/DDP/miR-493 and their paired control cells were constructed. Forced expression of miR-493 significantly enhanced the sensitivity of A549/DDP cells to cDDP-induced growth inhibition and apoptosis (Fig. 2a, b). Furthermore, to explore the functions of miR-493 in repair of cDDP-induced DNA damage, we evaluated the levels of H2AX phosphorylation on serine 139 (γ-H2AX) in lung cancer cells. cDDP can stimulate the foci formation of γ-H2AX, and γ-H2AX is the sensitive marker for DNA damage and DNA repair [20]. Here we carried out immunofluorescence staining to examine γ-H2AX foci formation. As shown in Fig. 2c, upon cDDP treatment, more γ-H2AX foci were observed in miR-493-overexpression cells compared with control cells. In addition, we validated whether miR-493 could play a role in tumor sensitivity to cDDP in vivo. A549 and A549/DDP cells transfected with either miR-493 or a scrambled control were transplanted into nude mice subcutaneously and developed into solid tumors in 7-10 days, and then 2 mg/kg cisplatin or equal volume saline were intraperitoneally injected every other day for 4 weeks. The tumor growth curves and tumor inhibited rate histogram were mapped. As shown in Fig. 3a and b, the tumor volume of miR-493-overexpressing xenografts decreased to a greater extent than that of control xenografts upon cDDP treatment, indicating a chemo-sensitizing effect of miR-493. Meanwhile, to investigate the cDDP-induced growth inhibition, we detected the expression of ki67, a marker for cell proliferation, in indicated nude mice tumor tissue. The data implied that Ki67 staining scores were lower in transfected miR-493 groups than controls. In brief, these data implied that miR-493 can markedly increase tumor cisplatin sensitivity and enhance the cisplatin induced growth inhibition in vitro and in vivo.

### miR-493 enhances the sensitivity of lung cancer cells to cisplatin by targeting TCRP1

**Fig. 4:**
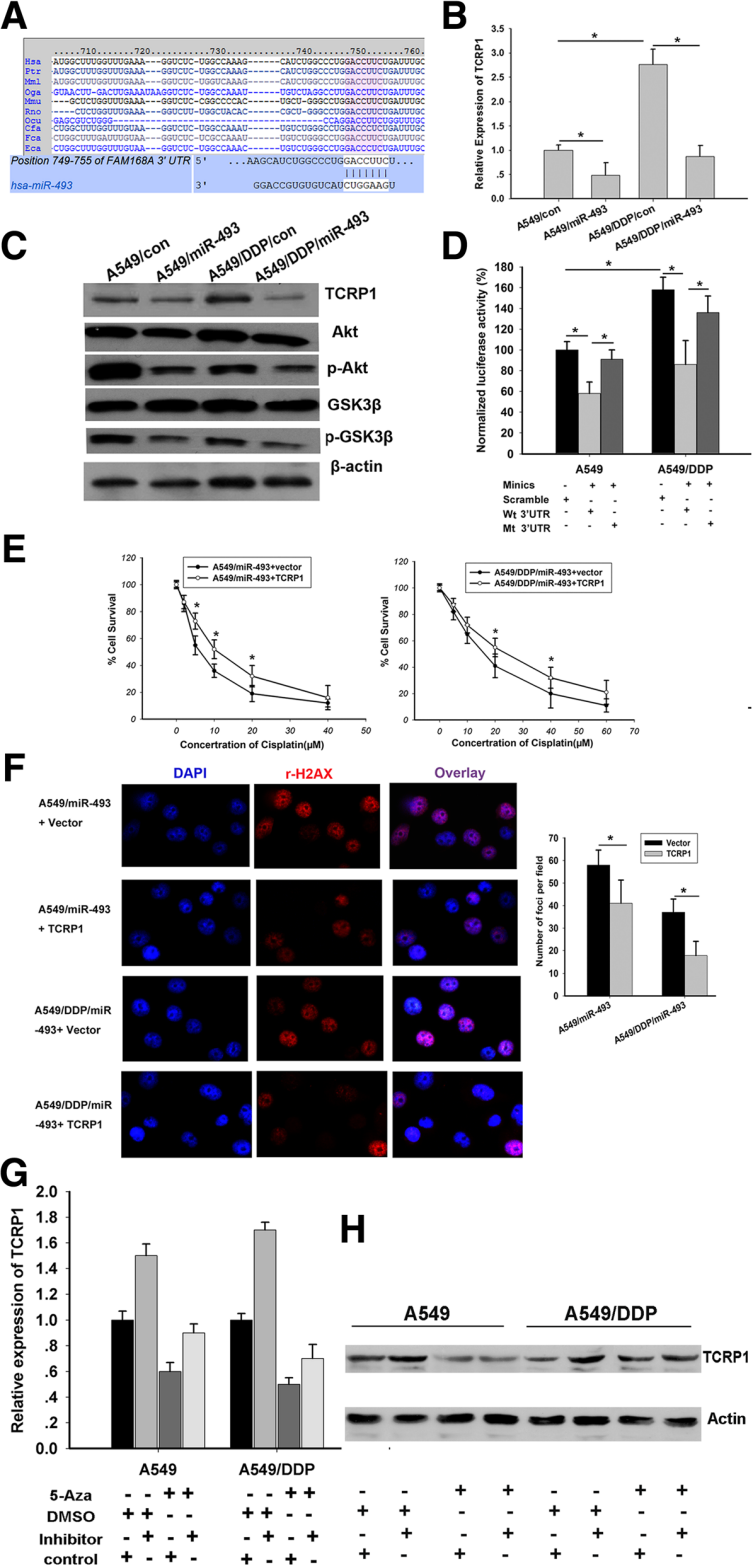
miR-493 enhences the sensitivity of lung cancer cells to cisplatin by targeting TCRP1. **a** Schematic of predicted miR-493 site in the 3’UTR of human TCRP1 mRNA, which broadly conserved among vertebrates. **b** miR-493 stable transfection reduces the TCRP1 mRNA levels. **c** Enforced expression miR-493 inhibits TCRP1 protein level and Akt signal pathway. **d** Luciferase assay of A549 and A549/DDP cells, which were co-transfected with miR-493 and a luciferase reporter containing full lengthTCRP1 3′-UTR (wt 3′-UTR) or a mutant (mut 3′-UTR) in which the nucleotides of the miR-493-binding site were mutated. An empty luciferase reporter construct was used as a negative control (scramble). The luciferase activities were measured 48 h post transfection. miR-493 markedly suppressed the luciferase activity in Luc-wt reporter constructs. The data are the means ± s.e.m. for separate transfections (*n* = 3). *P < 0.05 versus scramble. **e** MTS assay indicated that overexpression of exogenous TCRP1 (without 3’-UTR of TCRP1) rescued upon the sensitization induced by miR-493 in cells. **f** exogenous expression of TCRP1 attenuates DNA damage induced by miR-493 in lung cancer cells. Immunofluorescence staining was performed to examine γ-H2AX foci formation. γ-H2AX foci were counted in 5 random 200× micro fields under fluorescence microscope. Representative images of the assays are shown. Original magnification: ×200.Significant differences are indicated, (*, *P* < 0.05). **g**, **h** A549 or 549/DDP cells were treated with neither 2 uM 5-AZA or DMSO for 72 h, next treated with miR-493 inhibitor (RiboBio Co., Ltd) or scrambled control for 24 h, the mRNA and protein expression of TCRP1 were detected

We further investigated the mechanism by which miR-493 increases cisplatin sensitivity of lung cancer cells. The miRNA target prediction algorithm (TargetScan and microRNA.org) predicted that the 3′-UTRs of TCRP1 mRNA contain putative miR-493 binding sites (positions 749-755 of TCRP1 3’UTR, Fig. 4a). Our previous studies identified that TCRP1 plays an important role in cisplatin resistance [16, 18]. Then, we elucidate whether the miR-493 increases cisplatin sensitivity by repression of TCRP1. Western blotting and RT-PCR analysis revealed that expression of TCRP1 was dramatically decreased in miR-493-transfected lung cancer cells compared with controls (Fig. 4b, c). Meanwhile, dual-luciferase reporter analysis showed that miR-493 significantly inhibited the activity of firefly luciferase that carried wild type but not mutant 3′-UTR of TCRP1 (Fig. 4d). In addition, rescue experiments were performed by overexpressing the TCRP1-D3′- UTR vector (without an endogenous 3′- UTR) in cell expressing miR-493. The overexpression of TCRP1 attenuated the enhancement of sensitivity to cisplatin and reduction of DNA damage repair caused by miR-493(Fig. 4e, f). Moreover, in aforementioned paraffin-embedded mice xenografts tumor tissues, miR-493 and TCRP1 expression were detected by ISH or ICH, respectively. The staining scores indicated an inverse correlation between expression of miR-493 and that of TCRP1 (Fig. 3c). Our previous reports indicated that TCRP1 might mediate the sensitivity to cisplatin via Akt signal pathway [16, 21, 22]. Here, we checked the activation status of Akt signal pathway. The results implied that forced expression of miR-493 indeed inhibited the Akt pathway activation (Fig. 4c). Otherwise, In order to further validate the specific regulatory relationship between miR-493 and TCRP1, A549 or 549/DDP cells were treated with neither 2 uM 5-AZA or DMSO for 72 h, next treated with miR-493 inhibitor (RiboBio Co., Ltd., Guangzhou, China) or scrambled control for 24 h, and then checked the mRNA and protein expression of TCRP1. The data suggested that treatment with 5-Aza could significantly decrease the mRNA and protein levels of TCRP1. Whereas, miR-493 inhibitors could partly attenuate the down-regulation of TCRP1 (Fig. 4g, h). These observations suggest that miR-493 could specifically target TCRP1, which contribute to the effect on resistance to cisplatin.

### TCRP1 and miR-493 levels were associated with prognosis of lung cancers patients.

**Fig. 5:**
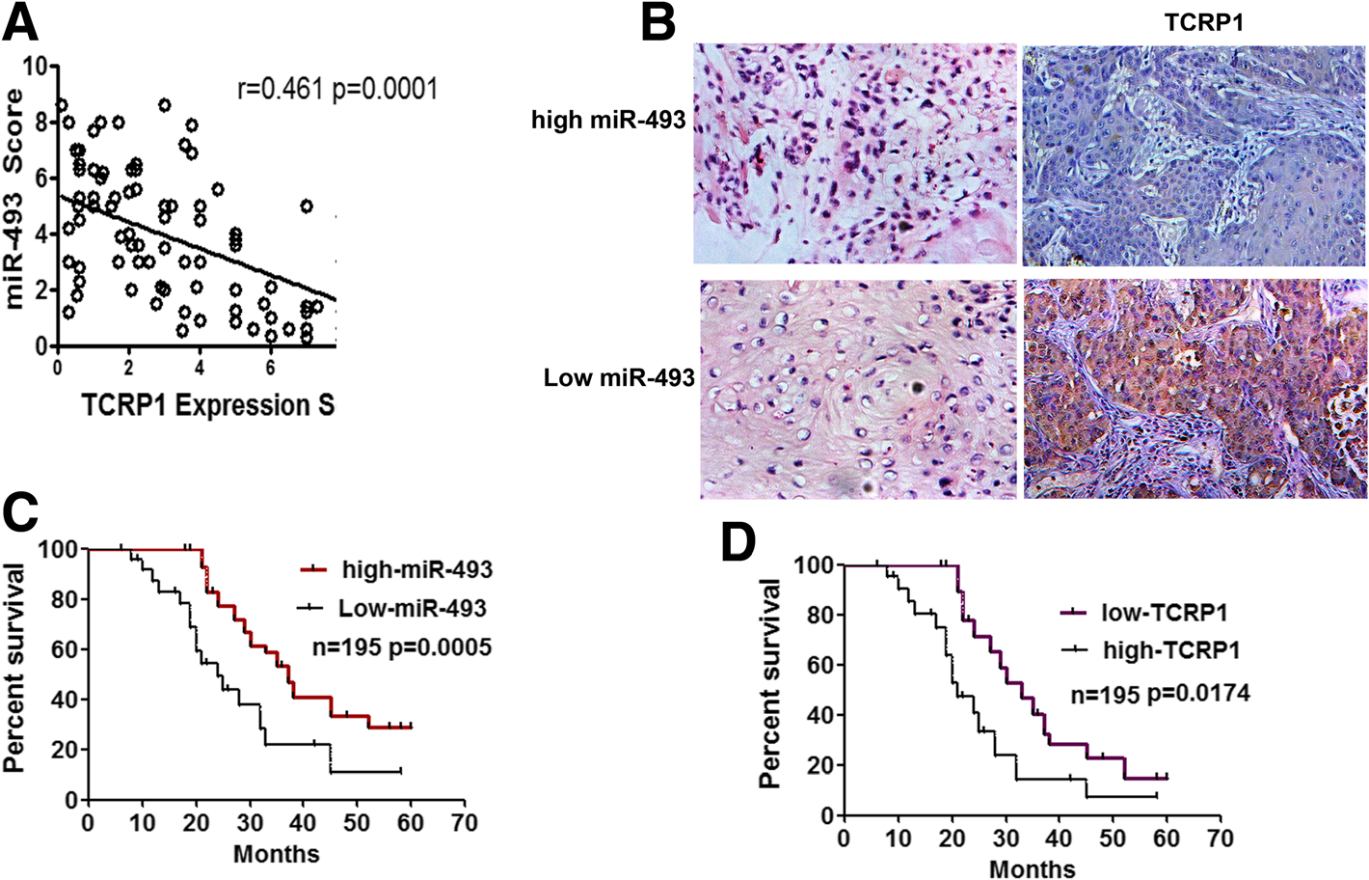
TCRP1 protein levels were inversely correlated with miR-493 levels and associated with prognosis of lung cancers patients. **a** miR-493 expression score was negatively correlated with TCRP1 expression score in lung cancer tissues. Spearman Rank test(*r* = 0.461, *p* = 0.0001). **b** Representative images of miR-493 expression detected by ISH (red staining as nucleus and blue staining as miR-493 level) and TCRP1 protein levels detected by immunohistochemical staining in lung cancer tissues (20×). **c**, **d** Kaplan-Meier analysis estimated overall survival according to the miR-493 expression and TCRP1 protein level

Given the regulatory relationship between miR-493 and TCRP1 identified in our cell culture studies, we focused whether the miR-493/TCRP1 might associate with the prognosis of lung cancer patients. The expression levels of miR-493 and TCRP1 were evaluated in clinical samples of lung cancer patients. We performed in situ hybridizations (ISH) to detect miR-493 and immunohistochemical staining to examine TCRP1 in the tissues from 195 lung cancer patients who received chemotherapy based on cDDP. Remarkably, ISH results demonstrated that 129(66.15%) lung cancer tissues exhibited relative low expression of miR-493. Among the 129 tissues, 109 of them (84.50%) showed relative high expression levels of TCRP1 (Fig. 5a and b). On the other hand, TCRP1 protein was highly expressed in only 15 (22.73%) of 66 lung cancer tissues with relative high expression of miR-493 (Fig. 5b), suggesting an inverse correlation between miR-493 and TCRP1 in lung cancers. To determine whether the dysregulation of miR-493 impacts the lung cancer phenotypes or clinical pathological features, we employed a correlation analysis and found that miR-493 expression level was not correlated with pathological parameters such as the clinical stage (Table 1). In addition, a Kaplan–Meier survival analysis was conducted using patient overall survival (OS) to analyze the significance of miR-493 further in terms of clinical prognosis. The results showed that lung cancer patients with low expression of miR-493 have a poorer prognosis for overall survival as compared to the patients with high expression of miR-493 (Fig. 5c). Interestingly, high protein levels of TCRP1 was also predictive for a worse overall survival in lung cancer patients (Fig. 5d). These data demonstrated a negative expression pattern between miR-493 and TCRP1, indicating that miR-493 might target TCRP1 in vivo as well. Our clinical studies support that the miR-493/ TCRP1 associates with the prognosis of lung cancer patients.

**Table 1:** Analysis of the correlation between expression of miR-493 in primary NSCLC and its clinicopathological parameters

clinicopathological rameters	Total cases	High expression	Low expression	*P*-value
Total patients	195	66	129	
Age (years)				0.365
≥60	117	36	81	
<60	78	30	48	
Sex				0.450
Male	135	42	93	
Female	60	24	36	
Stage (NSCLC)				0.215
I, II	114	51	63	
III+ IV	81	15	66	
Histology				0.511
SCC	75	30	45	
Adenocarcinoma	84	24	60	
Others	36	12	24	
Metastasis status				0.065
Metastasis	84	15	69	
No metastasis	111	51	60	

*NSCLC* nonsmall-cell lung cancer, *SCC* squamous cell carcinoma

## Discussion

In current study, we have shown that miR-493 might be a novel drug-resistant relative microRNA silenced in lung cancer due to the aberrant DNA methylation, highlighting DNA methylation as a mechanism for epigenetic silencing of miRNA genes during the development of acquired drug-resistance induced by chemotherapeutics. Furthermore, we have demonstrated miR-493 might function in lung cancer cells by regulating the expression of TCRP1, a resistant relative gene specifically to cisplatin. Recently, numerous reports have shown the critical role of epigenetic modifications in human cancers [23–26]. Altered epigenetic pattern such as the abnormal methylation of CpG islands at the gene promoter regions is one of the most common epigenetic alterations in cancer, affecting both coding and noncoding genes. The aberrant DNA methylation of the miRNA upstream DNA sequence plays a significant role in cancer progression including cell proliferation, migration and invasion, apoptosis, as well as therapy resistance [27, 28]. Although numerous DNA methylation-mediated epigenetic silencing of miRNAs has previously been demonstrated [29–31], the specific conditions of and mechanisms by which individual miRNA genes are silenced by methylation remain further described. Since gene-specific hypermethylation is an early and likely an initiating event in the process of tumor development [4–6], it will be of particular interest to identify miRNA genes sensitive to pathogenic DNA methylation, as well as the affected miRNA target genes in specific disease states. In addition, the molecular mechanisms of how chemotherapeutics causes an increase in DNA methylation are an important question to pursue in future studies.

Researchers have investigated the role of miR-493 in several cancers. The low expression levels of miR-493 have been detected, and are associated with tumor proliferation, angiogenesis and cancer metastasis [12–14, 32, 33], implying that miR-493 can serve as a tumor suppressor miRNA by negatively regulating cellular oncogenes. These are consistent with our previous studies of miR-493, in which MicroRNA-493 suppresses tumor growth and metastasis in lung cancer [15]. Furthermore, Tambe M and colleagues [33] found that miR-493-3p controls mitotic fidelity and cancer cells’ sensitivity to paclitaxel in ovarian and breast cancer. However, up to date, no substantial evidence supporting the causal link between suppression of miR-493 expression and epigenetic modification of the DNA sequence. Accordingly, here we have shown that not only miR-493 functions as a modulator of resistance to cisplatin in lung cancer cells, but also, for the first time, the aberrant methylation of promoter differential methylation region (DMR, +54 to +156) suppresses miR-493 expression in lung cancer.

In addition, several studies have shown that the inactivation of miR-493 has led to the overproduction of oncogenic RhoC and FZD4 that promotes cell migration and invasion in bladder cancer [13]; IGF1R that promotes colon cancer cells metastasis to liver [12]; and FUT4 that enhances the invasiveness and tumorigenicity in human breast cancer [14]. In this study, we also confirmed that TCRP1 (also known as FAM168A), a resistant relative gene in oral cancer and lung cancer is targeted regulated by miR-493. First, the re-expression of miR-493 in lung cancer cells down regulated the mRNA and protein expressions of TCRP1 (Fig. 4b-c) and increased the growth inhibition and apoptosis induced by cisplatin in vitro and in vivo (Fig. 2a-b, Fig. 3a-c,). Next, dual-luciferase reporter analysis showed that miR-493 significantly inhibited the activity of firefly luciferase that carried wild type but not mutant 3′-UTR of TCRP1 (Fig. 4d). Rescue experiments show that the overexpression of TCRP1 attenuated the enhancement of sensitivity to cisplatin and reduction of DNA damage repair caused by miR-493(Fig. 4e, f). Meanswhile, promoter hypomethylation by treatment with 5-Aza could elevate the expression of miR-493, which can decrease the expression of TCRP1; implying TCRP1 might be a direct target gene of miR-493 (Fig. 4g, h). A measurement of miR-493 and TCRP1 expression in 195 lung cancer tissue suggested an inverse correlation between miR-493 and TCRP1 in lung cancer patients (Fig. 5a). More important, a Kaplan–Meier survival analysis indicated that low expression of miR-493 and high expression of TCRP1 consistently were predictive for a worse overall survival in lung cancer patients (Fig. 5c-d). Tongue cancer resistance-associated protein 1 (TCRP1) was cloned by our group from a tongue cancer multi-drug resistance cell line Tca8113/PYM. In previous studies, we found that TCRP1 mediated a specific resistance to cisplatin in oral cancer cells, and functioned through activation of the PI3K/Akt/NF-κB signaling pathway and decreased cells apoptosis [16, 17]. Liu, et al. [18] found that TCRP1 regulates the level of Pol b by preventing its degradation, which increases DNA damage repair and reduces the apoptosis induced by cisplatin. Correspondingly, in this study, we also found that forced expression of miR-493 indeed inhibited the Akt pathway activation via decreasing TCRP1 expression.

## Conclusions

In summary, we demonstrated that silencing miR-493 through DNA methylation may have a substantial effect on gene expression. Our findings also revealed that miR-493 blocks the downstream targets gene TCRP1, thereby modulates the sensitivity to cisplatin in lung cancer. Enforced expression of miR-493 could partly reverse the resistant phenotype of lung cancer in vitro and in vivo. Meanwhile, miR-493 level is negatively correlated with TCRP1 expression and both expressions were correlated with overall survival in lung cancer patients. These findings suggest that miR-493 may serve as a predictive and potential therapeutic biomarker for overcoming the drug-resistance in lung cancer.

## Abbreviations

5-Aza-dC: 5-aza-deoxycytidine
ATCC: the American Type Culture Collection
BSP: Bisulfite sequencing PCR
GAPDH: Glyceraldehyde 3-phosphate dehydrogenase
ISH: In situ hybridization
MTS: [3-(4,5-diethylthiazol-2-yl)-5-(3-carboxymethoxyphenyl)-2-(4-sulfophenyl)-2H-etrazolium]
NSCLC: Non-small cell lung cancer
OD: Opticla Density
PYM: Pingyangmycin or bleomycin A5
qRT-PCR: Quantitative RT-PCR
TCRP1: Tongue cancer resistance-related protein1
γ-H2AX: Phosphorylated histone 2A variant X

## References

[1] Chen W, Zheng R, Baade PD, Zhang S, Zeng H, Bray F (2016). Cancer statistics in China, 2015. CA Cancer J Clin.

[2] Heng HH, Bremer SW, Stevens JB, Ye KJ, Liu G, Ye CJ (2009). Genetic and epigenetic heterogeneity in cancer: a genome-centric perspective. J Cell Physiol.

[3] Van Den Broeck A, Ozenne P, Eymin B, Gazzeri S (2010). Lung cancer: a modified epigenome. Cell Adhes Migr.

[4] Zochbauer-Muller S, Fong KM, Virmani AK, Geradts J, Gazdar AF, Minna JD (2001). Aberrant promoter methylation of multiple genes in non-small cell lung cancers. Cancer Res.

[5] Belinsky SA, Nikula KJ, Palmisano WA, Michels R, Saccomanno G, Gabrielson E (1998). Aberrant methylation of p16(INK4a) is an early event in lung cancer and a potential biomarker for early diagnosis. Proc Natl Acad Sci U S A.

[6] Wang YC, Lu YP, Tseng RC, Lin RK, Chang JW, Chen JT (2003). Inactivation of hMLH1 and hMSH2 by promoter methylation in primary non-small cell lung tumors and matched sputum samples. J Clin Invest.

[7] Wagner KW, Alam H, Dhar SS, Giri U, Li N, Wei Y (2013). KDM2A promotes lung tumorigenesis by epigenetically enhancing ERK1/2 signaling. J Clin Invest.

[8] Sarkar S, Horn G, Moulton K, Oza A, Byler S, Kokolus S, Longacre M (2013). Cancer development, progression, and therapy: an epigenetic overview. Int J Mol Sci.

[9] Wong KY, Yu L, Chim CS (2011). DNA methylation of tumor suppressor miRNA genes: a lesson from the miR-34 family. Epigenomics.

[10] Yan JW, Lin JS, He XX (2014). The emerging role of miR-375 in cancer. Int J Cancer.

[11] Xi S, Xu H, Shan J, Tao Y, Hong JA, Inchauste S (2013). Cigarette smoke mediates epigenetic repression of miR-487b during pulmonary carcinogenesis. J Clin Invest.

[12] Okamoto K, Ishiguro T, Midorikawa Y, Ohata H, Izumiya M, Tsuchiya N (2012). miR-493 induction during carcinogenesis blocks metastatic settlement of colon cancer cells in liver. EMBO J.

[13] Ueno K, Hirata H, Majid S, Yamamura S, Shahryari V, Tabatabai ZL (2012). Tumor suppressor microRNA-493 decreases cell motility and migration ability in human bladder cancer cells by downregulating RhoC and FZD4. Mol Cancer Ther.

[14] Sakai H, Sato A, Aihara Y, Ikarashi Y, Midorikawa Y, Kracht M (2014). MKK7 mediates miR-493-dependent suppression of liver metastasis of colon cancer cells. Cancer Sci.

[15] Gu Y, Cheng Y, Song Y, Zhang Z, Deng M, Wang C (2014). MicroRNA-493 suppresses tumor growth, invasion and metastasis of lung cancer by regulating E2F1. PLoS One.

[16] Gu Y, Fan S, Xiong Y, Peng B, Zheng G, Yu Y (2011). Cloning and functional characterization of TCRP1, a novel gene mediating resistance to cisplatin in an oral squamous cell carcinoma cell line. FEBS Lett.

[17] Peng B, Gu Y, Xiong Y, Zheng G, He Z (2012). Microarray-assisted pathway analysis identifies MT1X & NFkappaB as mediators of TCRP1-associated resistance to cisplatin in oral squamous cell carcinoma. PLoS One.

[18] Liu X, Wang C, Gu Y, Zhang Z, Zheng G, He Z (2015). TCRP1 contributes to cisplatin resistance by preventing Pol beta degradation in lung cancer cells. Mol Cell Biochem.

[19] Liu X, Feng M, Zheng G, Gu Y, Wang C, He Z (2017). TCRP1 expression is associated with platinum sensitivity in human lung and ovarian cancer cells. Oncol Lett.

[20] Olive PL, Banath JP (2009). Kinetics of H2AX phosphorylation after exposure to cisplatin. Cytometry B Clin Cytom.

[21] Gu Y, Fan S, Liu B, Zheng G, Yu Y, Ouyang Y, He Z (2011). TCRP1 promotes radioresistance of oral squamous cell carcinoma cells via Akt signal pathway. Mol Cell Biochem.

[22] Wang C, Liu H, Qiu Q, Zhang Z, Gu Y, He Z (2017). TCRP1 promotes NIH/3T3 cell transformation by over-activating PDK1 and AKT1. Oncogene.

[23] Lv JF, Hu L, Zhuo W, Zhang CM, Zhou HH, Fan L (2016). Epigenetic alternations and cancer chemotherapy response. Cancer Chemother Pharmacol.

[24] Harada K, Baba Y, Ishimoto T, Kosumi K, Tokunaga R, Izumi D (2015). Suppressor microRNA-145 is epigenetically regulated by promoter Hypermethylation in esophageal Squamous cell carcinoma. Anticancer Res.

[25] Wang S, Zhang R, Claret FX, Yang H (2014). Involvement of microRNA-24 and DNA methylation in resistance of nasopharyngeal carcinoma to ionizing radiation. Mol Cancer Ther.

[26] Liloglou T, Bediaga NG, Brown BR, Field JK, Davies MP (2014). Epigenetic biomarkers in lung cancer. Cancer Lett.

[27] Jones PA, Baylin SB (2007). The epigenomics of cancer. Cell.

[28] Ellis L, Atadja PW, Johnstone RW (2009). Epigenetics in cancer: targeting chromatin modifications. Mol Cancer Ther.

[29] Toll A, Salgado R, Espinet B, Diaz-Lagares A, Hernandez-Ruiz E, Andrades E (2016). MiR-204 silencing in intraepithelial to invasive cutaneous squamous cell carcinoma progression. Mol Cancer.

[30] Kim YH, Lee WK, Lee EB, Son JW, Kim DS, Park JY. Combined Effect of Metastasis-Related MicroRNA, miR-34 and miR-124 Family, Methylation on Prognosis of Non-Small-Cell Lung Cancer. Clin Lung Cancer. 2016;18(1):e13–e20. 10.1016/j.cllc.2016.06.005 27444357

[31] Tellez CS, Juri DE, Do K, Picchi MA, Wang T, Liu G (2016). miR-196b is epigenetically silenced during the premalignant stage of lung carcinogenesis. Cancer Res.

[32] Zhao L, Feng X, Song X, Zhou H, Zhao Y, Cheng L, Jia L (2016). miR-493-5p attenuates the invasiveness and tumorigenicity in human breast cancer by targeting FUT4. Oncol Rep.

[33] Tambe M, Pruikkonen S, Maki-Jouppila J, Chen P, Elgaaen BV, Straume AH (2016). Novel Mad2-targeting miR-493-3p controls mitotic fidelity and cancer cells' sensitivity to paclitaxel. Oncotarget.

